# Ichthyol Internally in Psoriasis

**Published:** 1914-08

**Authors:** 


**Ichthyol Internally in Psoriasis.** A. Rose of N. Y., Merck's Archives, May, 1914, claims goods results and describes cases,

